# Reply to: Quantum mechanical rules for observed observers and the consistency of quantum theory

**DOI:** 10.1038/s41467-024-47172-0

**Published:** 2024-04-09

**Authors:** Lídia del Rio, Renato Renner

**Affiliations:** Institute for Theoretical Physics, ETH Zurich, Zurich, Switzerland

**Keywords:** Quantum mechanics, Theoretical physics

**replying to** A. P. Polychronakos *Nature Communications* 10.1038/s41467-024-47170-2 (2024)

## Wigner’s friend experiment

**Fig. 1:**
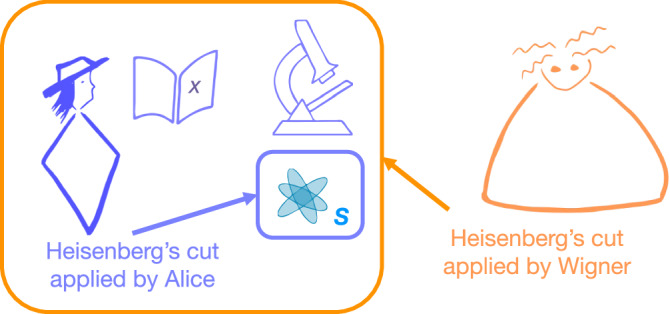
Wigner’s friend experiment. The experiment concerns two quantum mechanics, Alice and Wigner. Alice measures a system *S* and records an outcome *x*. Alice applies the Heisenberg cut around system *S*: she treats *S* as a quantum system but regards herself, her notebook, and the outcome *x* as classical. Wigner analyses the situation from the outside, applying the Heisenberg cut around Alice’s entire isolated lab: he models Alice, her notebook, her measurement instruments, and everything else in her lab as quantum systems undergoing a global reversible evolution.

In 1967, Eugene Wigner proposed a thought experiment to test the range of validity of quantum theory^1^. The experiment features two agents, Wigner and his friend, whom we call Alice (Fig. 1). Alice works in a perfectly isolated lab, and her task is to measure an observable *X* of a quantum system *S*. Wigner’s task is to analyse the same experiment from the outside, treating Alice’s entire lab as a quantum system. Crucial to this setup is that, when applying quantum theory, the two agents split the world differently into quantum and classical parts—i.e., they choose different ‘Heisenberg cuts’^2^. Alice only models *S* as a quantum system and treats the outcome of her measurement *X* as part of the classical domain, yielding a definite value, *x*. In contrast, Wigner models Alice’s entire lab as part of the quantum domain, including Alice herself and her memory of the measurement outcome. Hence, for Wigner, Alice’s measurement is a reversible entangling operation between her and *S*, and *x* has no definite value. Alice and Wigner’s conclusions regarding *x* are thus different, although, at this point, they are not strictly contradictory.

## The FR experiment

In 2018, Daniela Frauchiger and one of us (Renner) proposed an extension of Wigner’s thought experiment, often referred to as the ‘FR experiment’^3^ (see also^4^ for a similar proposal). It involves a group of four agents tasked with making predictions about each other’s measurements. Crucially, each agent applies the Heisenberg cut subjectively and may choose to model some of the other agents as quantum systems. All agents share the same initial information about the experimental setting and protocol, but during the actual run of the experiment, each agent may have access to additional data based on their local observations. Each agent analyses the experiment from their perspective using the same reasoning rules described by (Q), (C), and (S) below. The key insight of the experiment is that the agents reach contradictory conclusions; this result was framed as the no-go theorem^3^ restated here. For an in-depth analysis of the FR experiment in the light of different interpretations of quantum theory, we refer to^5,6^.

### Theorem 1

^3^. No physical theory where it is possible to model the FR experiment is compatible with the reasoning rules (Q), (C), and (S).

Considered individually, each reasoning rule appears intuitive and unproblematic; nonetheless, Theorem 1 asserts that they are contradictory. For simplicity, we elucidate these reasoning rules by describing their use by an agent, Alice, who is deriving statements about the outcome *x* of a measurement specified by an observable *X*.

(Q) *Validity of quantum theory at the relevant scales:* Suppose that the observable *X* is defined on a quantum system *S* around Alice (i.e., Alice is not herself part of *S*). Alice may then apply the standard formalism of quantum theory to describe *S* and calculate the probabilities for the potential measurement outcomes. In particular, if this analysis yields that the outcome is *x* with probability 1, Alice can conclude “I am certain that *X* = *x*.” (For concreteness, the ‘standard formalism’ can be the four quantum postulates of Nielsen & Chuang^7^, Section 2.2, applied to the system *S* and its subsystems).
(C) *Consistency among agents:* Let Bob be another agent who reasons about the same measurement *X*. If Alice has deduced, “I am certain that Bob has concluded that he is certain that *X* = *x*” then Alice can conclude, “I am certain that *X* = *x*.”
(S) *Single outcomes:* If Alice has concluded both “I am certain that *X* = *x*” and “I am certain that $X={x}^{{\prime} }$” for $x\ne {x}^{{\prime} }$ then she considers that a contradiction.

## A simple experiment to test reasoning rules

**Fig. 2:**
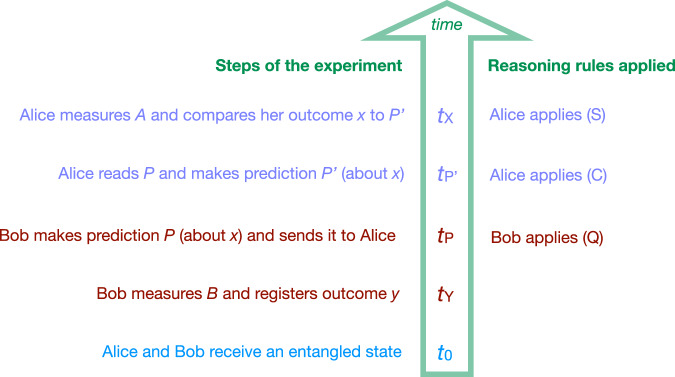
Applying reasoning rules to the Learned Prediction Experiment. If we omit Wigner’s measurement, the analysis of this experiment is straightforward. For example, if Bob observes *Y* = 1, he can use reasoning rule (Q) to infer the prediction *P* = "I am certain that *X* = 1.” Upon receiving and reading *P*, Alice may say “I am certain that Bob is certain that *X* = 1.” Using (C) Alice immediately arrives at ${P}^{{\prime} }=$"I am certain that *X* = 1.” Finally, (S) demands that the outcome of Alice’s measurement must indeed match her prediction ${P}^{{\prime} }$.

The idea behind rules (Q), (C), and (S) is that they correspond to the building blocks of reasoning that physicists naturally employ to analyze standard experiments. To see this, we introduce a simple experimental setup, which we term the ‘Learned Prediction Experiment’ (Box 1): An agent, Alice, learns a prediction from another agent, Bob, where both agents use the reasoning rules above, as shown in Fig. 2. An addition relevant to the later discussion is that a third agent, Wigner, may measure Bob’s lab at some point in the experiment. As we will see, different proposals to circumvent Theorem 1 will also lead to different conclusions about this experiment.

Box 1 Learned Prediction Experiment

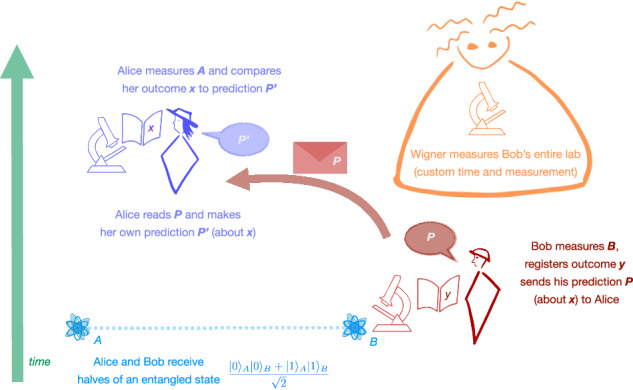

- *t*_0_: Alice and Bob receive qubits *A* and *B* respectively, jointly prepared in an entangled Bell state $({\left\vert 0\right\rangle }_{A}\otimes {\left\vert 0\right\rangle }_{B}+{\left\vert 1\right\rangle }_{A}\otimes {\left\vert 1\right\rangle }_{B})\,/\sqrt{2}$.
- *t*_*Y*_: Bob measures qubit *B* in the computational basis $\{\left\vert 0\right\rangle,\left\vert 1\right\rangle \}$ and registers his outcome *y*.
- *t*_*P*_: Bob makes a prediction *P* for the outcome *x* of Alice’s measurement at *t*_*X*_ (see ahead) and communicates *P* to Alice.
- ${t}_{{P}^{{\prime} }}$: Alice receives *P* and infers from this a prediction ${P}^{{\prime} }$ for the outcome *x* of her measurement at *t*_*X*_.
- *t*_*X*_: Alice measures qubit *A* in the computational basis $\{\left\vert 0\right\rangle,\left\vert 1\right\rangle \}$ and compares her outcome *x* to her prediction ${P}^{{\prime} }$.
- *t*_*W*_: Wigner carries out a measurement of his choice on Bob’s lab (which may include qubit *B*, Bob’s memory, measurement instruments, and environmental degrees of freedom).

The first four steps occur at fixed times ordered as ${t}_{0} < \, {t}_{Y} < \, {t}_{P} < \, {t}_{{P}^{{\prime} }} < \, {t}_{X}$. Wigner’s measurement occurs at a time *t*_*W*_, which is also fixed but customizable. All agents are initially provided with a description of this protocol, including the timing of the steps. Furthermore, they know that all agents use the same set of reasoning rules to obtain their predictions.

## Criticism of Theorem 1

A large number of recent works have criticised Theorem 1—not its technical statement or proof, but rather the nature of its assumptions, reasoning rules (Q), (C), and (S). This, of course, is precisely the point of the no-go theorem—it asserts that the combination of these reasoning rules leads to contradictions. Nonetheless, Theorem 1 is only of interest if the reasoning rules (Q), (C), and (S) accurately capture the way we reason about physical experiments. Works criticising theorem typically claim this is not true for some of these reasoning rules.

To warm up, we illustrate this with a common criticism, which was eloquently summarized by Scott Aaronson as ‘It’s hard to think when someone Hadamards your brain’^8^. The term ‘Hadamard’ refers here to a particularly destructive measurement, applied to an agent’s lab, which is incompatible with the computational basis we would use to describe how the agent processes and stores information when reasoning. (This is also sometimes called a ‘Bell measurement’ or ‘cat measurement’^9^ because it often corresponds to a measurement in the Bell basis of the agent’s memory and the system they observed.) The argument may be expressed in terms of a restriction on using rule (C).

*Restriction 1*. Reasoning rule (C) cannot be applied to predictions that Bob made after his memory was subjected to a destructive measurement.

**Fig. 3:**
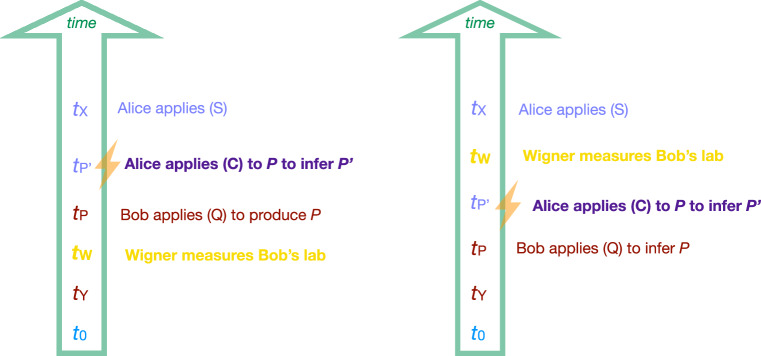
The Learned Prediction Experiment under different restrictions. Left: If Wigner measures Bob’s lab before Bob produces his prediction (*t*_*W*_ < *t*_*P*_), Restriction 1^8^ forbids Alice from applying reasoning rule (C). This restriction ensures that agents do not rely on predictions computed by a malfunctioning agent. (In the FR thought experiment, this requirement is taken care of by the appropriate timing of the protocol steps.) Right: Restriction 2^9^ forbids Alice from applying (C) (as in Fig. 2) even if Wigner measures Bob’s lab after Bob sends his prediction.

In the case of the Learned Prediction Experiment, and assuming Wigner’s measurement is indeed destructive, Restriction 1 means that the use of (C) should be forbidden if *t*_*W*_ ≤ *t*_*P*_ (Fig. 3).

## Why Restriction 1 is sensible but irrelevant

We agree with this reasoning. In fact, Restriction 1 is already taken into account implicitly by the assumption that the agents all apply the same reasoning rules. Measurements that are so destructive that they disturb the agent’s reasoning process are thus ruled out. More importantly, however, Restriction 1 is irrelevant in the case of the FR thought experiment. While (potentially) destructive measurements are applied to agents, the timing of these measurements is such that an agent never needs to make or communicate a prediction after having been subjected to such a measurement. Hence, Restriction 1, while absolutely justified, does not resolve the contradiction between the reasoning rules (Q), (C), and (S).

## A stronger restriction

In a recent comment^9^, Alexios Polychronakos analyses the FR thought experiment using an approach termed ‘unitary quantum mechanics’, which basically consists of putting the Heisenberg cut at the outside of the entire experiment. Technically, such an analysis corresponds to the one presented in^10^ or^11^ (the latter employs Bohmian mechanics; see the Supplementary Information for more details as well as a clarification of their claim that Theorem 1 is invalid). Motivated by this analysis, the author argues that if agents reason based on information held by other agents, along the lines of rule (C), then they arrive at invalid predictions—in agreement with Theorem 1^3^. Because Restriction 1 does not rule out this use of rule (C), he suggests extending the restriction to destructive measurements that lie in the future. In the spirit of Aaronson’s slogan, this suggestion may be phrased as ‘It’s hard to think when someone will later Hadamard your brain.’

*Restriction 2*. Reasoning rule (C) cannot be applied to predictions that Bob made if his memory is subjected to a destructive measurement — even if that measurement lies in the future.

Applied to the Simple Prediction Experiment, Restriction 2 would imply that our analysis above is invalid even if Wigner measures Bob in the far future, i.e., after Bob has sent his prediction *P* to Alice, and possibly also after Alice has completed her measurement to verify the prediction (Fig. 3). In fact, the proposal by Polychronakos goes even one step further and similarly restricts the use of rule (Q). The author concludes that, equipped with these additional restrictions, the reasoning rules no longer yield a contradiction in the setting of the FR thought experiment.

We agree with this conclusion but would like to point out that such restrictions on the agents’ reasoning entail various problems, which we detail in the following (see also the Supplementary Information).

### Objection 1

**Restrictions 1 and 2 are ambiguous**. In the formulation of Restrictions 1 and 2 above, we used the term ‘destructive measurement’, which we believe is in the spirit of^8^ and^9^ (in these works, the terms ‘Hadamard’ and ‘cat measurement’ are used). But to make this unambiguous, it is necessary to characterise which measurements count as ‘destructive’. Clearly, the particular measurements on the friends’ labs in the FR experiment must be treated as destructive, but this is not sufficient; it would be easy to find variations of the FR experiment that also lead to contradictory conclusions with measurements that are just slightly rotated from the original ones. In that case, the safety induced by Restriction 2 would not be robust under small changes. On the other hand, if a proposal would extend the constraint to all settings where Alice’s brain will be under any measurement, it would implicitly rule out even all classical logical reasoning used today—because our inferences are stored in physical memories that will eventually interact with their environments. It is unclear whether there is any natural boundary between these extremes that avoids either issue.

### Objection 2

**Physical justification of Restriction 2 requires signalling**. The constraint imposed by Restriction 2 on using reasoning rule (C) seems unnecessarily strict when applied to settings like our Learned Prediction Experiment. If Wigner measures Bob after Bob communicates his prediction *P* to Alice, one would not expect this to render *P* invalid. This intuition may be verified by modelling Bob as a quantum system that outputs *P*. The non-signalling property of quantum theory then implies that a measurement performed by Wigner on Bob at time *t*_*W*_ > *t*_*X*_ cannot be noticed by Alice at time *t*_*X*_.

Restriction 2 is just a constraint on the applicability of a reasoning rule and hence does not imply signalling per se. However, if Restriction 2 was physically justified in the sense of having a physical origin, then the use of rule (C) without this restriction should sometimes lead to wrong predictions in experiments. To illustrate this, consider the Learned Prediction Experiment. If Restriction 2 was necessary here, the prediction ${P}^{{\prime} }$, computed by Alice using rule (C), would sometimes be wrong when Wigner measures Bob’s lab at a time *t*_*W*_ > *t*_*X*_. Because Alice can verify her prediction at time *t*_*X*_, this would violate the non-signalling principle: Wigner, by measuring or not measuring at time *t*_*W*_, could send a signal to Alice at time *t*_*X*_, into the past. In this sense, a physical justification for Restriction 2 is incompatible with the non-signalling principle.

### Objection 3

**Restrictions on (C) impair reasoning**. Rule (C) allows agents to combine and compress information, which facilitates processing and prediction-making. Restrictions on the use of this rule may thus impair reasoning even in standard settings, where agents typically hold only partial information about the physical setup and must apply (C) to piece together their local bits of knowledge.

In our Learned Prediction Experiment, we assumed that all agents were provided with a full description of the experimental protocol. This allows Alice, in principle, to reach her prediction ${P}^{{\prime} }$ without applying (C): Alice may reverse-engineer Bob’s reasoning to infer his outcome *y* from his prediction *P*, which was communicated to her. Knowing *y* she may then employ (Q) to come up with ${P}^{{\prime} }$. However, this strategy for avoiding the use of (C) would not be available to Alice if her knowledge about the experimental setup was partial. An example of this would be a variant of the experiment where Alice’s initial information consists of a description of her local setup only, so that she cannot simulate Bob.

That agents have partial information is common in real-world examples and particularly dramatic in cryptography scenarios. For example, in quantum key distribution protocols, without (C), Alice cannot make the logical step from “Bob publicly announced that his measurement basis was *X*” to “I know that Bob’s measurement basis was *X*.” It is unclear whether Alice and Bob, who in the setting trust each other but otherwise are embedded in an environment controlled by an adversary, can obtain any security guarantee for the distributed key without applying (C)^12^.

But even in the special case where all agents have full information about the global setup, so that (C) could be substituted by (Q), this comes with a complexity overhead. An agent who wants to incorporate knowledge communicated by another agent would need to simulate that agent as a quantum system. In general network scenarios with *N* agents whose individual predictions may depend on chains of reasoning across several agents, the (classical) memory required for this scales exponentially with *N*^13^.

## Desiderata for resolutions of the paradox

We note that various other suggestions have been made in the recent literature to evade the contradiction in the FR experiment (see^14–20^ for examples). Similarly to Restrictions 1 and 2 above, they postulate constraints on the reasoning rules, notably rules (Q) and (C). Likely, the objections discussed above are also relevant to them. More generally, any proposal to resolve the paradox faces the challenge of finding a fine balance. If the restriction on the reasoning rules is too moderate, they may still yield contradictory conclusions when applied to thought experiments like FR. Conversely, if the reasoning rules are constrained too much, their usability in everyday situations may be affected. To foster further research, we propose a list of desiderata for proposals for modified reasoning rules.

1. *Clear:* The proposed reasoning rules should be specified unambiguously (see Objection 1).
2. *Usable:* The proposed reasoning rules should be usable by an agent who is a physical system embedded in the physical world and who has only partial information about the world. In particular, the reasoning rules can only depend on information that is physically available to the agent and can be processed with the agent’s physical resources (see Objection 3).
3. *Falsifiable:* The proposed reasoning rules should reproduce the predictions of quantum theory in all regimes that have been experimentally tested, including scenarios where individual agents have only partial information about the overall setup. In particular, any data produced by a realistic experiment that would falsify quantum theory should also falsify the reasoning rules.
4. *Consistent:* The proposed reasoning rules should apply to any experiment describable within the standard formalism of quantum theory, including thought experiments such as the Wigner’s friend or the FR experiment, and should not yield contradictions.
5. *Physical:* The proposed reasoning rules should be physically justifiable; in particular, they should avoid the violation of basic physical principles (see Objection 2).

## Outlook

We urge those who propose modified reasoning rules to circumvent Theorem 1 not to be discouraged by the objections presented here. This is a recent and novel problem, and it is only natural that the appropriate tools to tackle it have not yet been developed. To study and test reasoning rules in view of the desiderata listed above, we leave the reader with two suggestions for such tools. For computational tests, the free software package for quantum thought experiments developed by Nurgalieva, Mathis and ourselves^13^ allows a user to formulate bespoke reasoning rules in a computer-readable manner and test them in different experimental settings: the software outputs the predictions of different agents and whether they are contradictory. For a theoretical analysis of Wigner’s friend-type experiments, a promising approach is the framework of Vilasini and Woods^21^ (see Supplementary Information), which enforces an explicit specification of the choice of the Heisenberg cut by the different agents.

### Supplementary information

Supplementary Information

## Data Availability

No data sets were generated or analysed during the current study.
