# Publisher Correction: Reply to: Quantum mechanical rules for observed observers and the consistency of quantum theory

**DOI:** 10.1038/s41467-024-47826-z

**Published:** 2024-04-22

**Authors:** Lídia del Rio, Renato Renner

**Affiliations:** Institute for Theoretical Physics, ETH Zurich, Zurich, Switzerland

**Keywords:** Quantum mechanics, Theoretical physics

Correction to: *Nature Communications* 10.1038/s41467-024-47172-0, published online 09 April 2024

In this article, the reasoning rule ‘(R) Consistency among agents…” in Theorem 1 should have read: ‘(C) Consistency among agents…’. The original article has been corrected.

