# Supplementary material for: Reply to: Quantum mechanical rules for observed observers and the consistency of quantum theory
Source: Nat Commun. 2024 Apr 9;15:3024. doi: 10.1038/s41467-024-47172-0 (PMC11004133; doi:10.1038/s41467-024-47172-0)
Supplement: Supplementary file 1 — Supplementary Information [file 41467_2024_47172_MOESM1_ESM.pdf]

Supplementary Information

Reply to: Quantum mechanical rules for observed observers and the consistency of quantum theory

Lídia del Rio* and Renato Renner†

Institute for Theoretical Physics, ETH Zurich, 8093 Zurich, Switzerland

Detailed description of the experiment

We can make some steps of the Learned Prediction Experiment more explicit, for example, by encoding Bob's message in a quantum state and specifying Wigner's measurement.

Experiment 1 (Learned Prediction Experiment)**.** *The experiment consists of three agents, Alice, Bob, and Wigner, two entangled qubits A and B (which Alice and Bob will measure in the computational basis $\{|0\rangle, |1\rangle\}$), and a qutrit P (equipped with a basis $\{|0\rangle, |1\rangle, |\perp\rangle\}$, which will encode Bob's prediction). The experimental protocol is as follows:*

t_0: *Alice receives A, and Bob receives P and B, prepared in joint quantum state*

$$|\psi\rangle_{ABP} = \frac{|0\rangle_A|0\rangle_B + |1\rangle_A|1\rangle_B}{\sqrt{2}} \otimes |\perp\rangle_P. \tag{1}$$

t_Y: *Bob measures his qubit B in the computational basis, and registers his outcome Y in his lab.*

t_P: *Bob reasons about Alice's measurement of A, and makes a prediction for the value of her outcome X. He encodes his prediction in the state of P by rotating it to $|0\rangle_P$ or $|1\rangle_P$ if his prediction is "Alice will obtain $X = 0$" or "Alice will obtain $X = 1$" respectively, and leaving it unchanged as $|\perp\rangle_P$ if his conclusion is "I cannot predict the outcome of Alice's measurement with certainty." He sends P to Alice.*

$t_{P'}$: *Alice receives P and measures it in the computational basis. Based on her outcome, Alice reasons about Bob's prediction and tries to make a prediction for her future outcome X. She stores this prediction P' in her lab.*

t_X: *Alice measures her first qubit A in the computational basis and registers her outcome X in her lab.*

t_W: *Wigner performs a measurement $\mathcal{M}$ on Bob's lab L, which includes qubit B, registering his outcome W.*

The following parameters can be customised: the spacetime positions of the different steps of the experiment, subject to the constraint that their partial ordering is $t_0 < t_Y < t_P < t_{P'} < t_X$; a model of Bob's memory and lab, specified as a quantum system L, and a description of how Bob physically

*lidia@squids.ch

†renner@ethz.ch

implements local measurements and reasoning steps [1]; and Wigner's choice of measurement, represented by an arbitrary projective measurement $\mathcal{M}$ on Bob's lab (or, more generally, the TPCPM that implements Wigner's measurement).

All agents agree on an arbitrary reference frame for spacetime and bases of all relevant Hilbert spaces, which remain constant throughout the experiment. All spacetime positions are relative to this reference frame, and the partial order $<$ is inherited from the chosen spacetime structure (for example, the standard causal ordering induced by special relativity). We assume that all agents know all the steps of the experiment and customisable parameters, unless otherwise indicated.

Applying the FR reasoning rules. Without Wigner's measurement ($\mathcal{M} = \{\mathrm{id}_L\}$), the analysis of the experiment is straightforward. All agents know that since qubits A and B are entangled in a Bell state, their outcomes will be perfectly correlated, and Bob will predict at time t_P that $X = Y$. Concretely, if he obtained outcome $Y = 0$ at t_Y, he predicts that Alice will obtain $X = 0$ and encodes this prediction in state $|0\rangle_P$; similarly, if he obtained $Y = 1$, he predicts that $X = 1$, and encodes his prediction in state $|1\rangle_P$. He applies assumption (Q) for this.

At time $t_{P'}$, Alice measures register P with Bob's prediction and obtains an outcome p. Unless $p = \perp$, she reasons that "at time t_P, Bob predicted that my future outcome would be $X = p$ with certainty." Alice can then apply rule (C) to reason "since I know that Bob predicted that $X = p$ with certainty, I can predict that my future outcome will be $X = p$ with certainty." In other words, Alice promotes Bob's prediction to her own: "I am certain that $X = p$."

At time t_X, Alice measures A and observes a value $X = x$, which allows her to make another statement: "I am certain that $X = x$." Now, for Alice not to arrive at a contradiction according to rule (S), it must be the case that $x = p$, i.e., Alice's measurement outcome x must agree with the prediction p she received from Bob.

Remark. For the analysis above, we have considered the case where Wigner's measurement is not applied, or where it is applied only after t_P, i.e., after Bob has communicated register P to Alice. If it was applied earlier, it could have disturbed Bob's reasoning process, and our general assumption that all agents apply the stated reasoning rules correctly is no longer valid, prohibiting Alice from employing (C) when reasoning about Bob's prediction P (see the discussion of Restriction 1 in the main text).

Objections to Restriction 2

Details on Objection 1: Restrictions 1 and 2 are ambiguous. In real life, all our brains and memories will eventually interact with our environments, and the local action of those interactions can be formalised as a non-unitary map, which can *always* be interpreted as a noisy quantum measurement — whether it is an intentional measurement by an outside agent or just standard dissipation. Proposals such as [2] neglect to specify precisely for what class of measurements we should restrict (C). If it is just for perfect 'cat measurements' (measurements in the Bell basis), as [2] seems to suggest, then it would be easy to find variations of the FR experiment that also lead to contradictory conclusions with measurements that are just slightly rotated from the original ones. In that case, the safety induced by the restriction would not be robust under small changes. On the other hand, if these proposals want to restrict (C) to all settings where Bob's brain will be under any measurement, they implicitly rule out even all classical logical reasoning used today — because our inferences are stored in memories that will eventually interact with their environments. It is unclear whether there is any natural boundary between these extremes that avoids either issue.

Details on Objection 2: Physical justification of Restriction 2 requires signalling. A natural response to any statement of the form 'You are not allowed to apply that reasoning strategy in this setting' is 'So what would happen if I did? Can you prove that I would reach incorrect conclusions?' In this case, one could say, 'You cannot apply (C) in the FR experiment, otherwise we show that you will reach a contradiction', but Polychronakos' restriction (and other proposals) extends to settings like

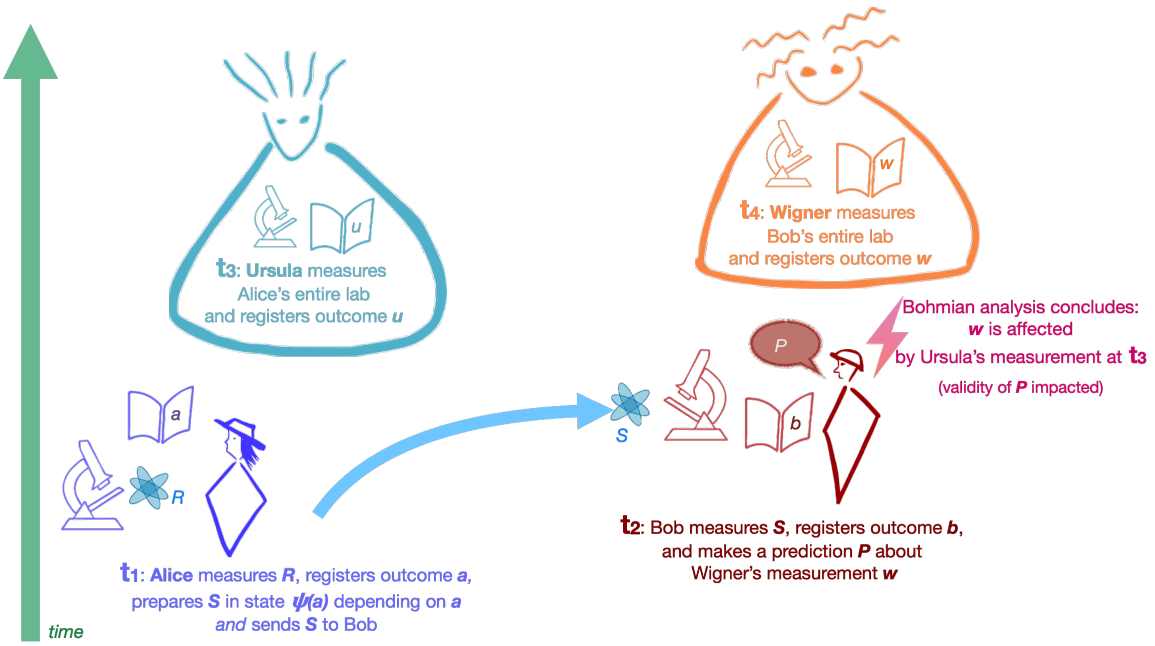

Supplementary Figure 1: **FR experiment.** Basic illustration of the FR experiment, without details of the measurement performed, states prepared, or reasoning steps, which have been extensively covered elsewhere [3, 4, 5, 1]. The key point is that a Bohmian analysis of the experiment says that Ursula's measurement of Alice at t_3 influences Wigner's outcome w at t_4. Note that after Alice sends S to Bob, we can space-like separate Bob from Alice. Therefore, Ursula's measurement influence on Wigner's outcome (and on the validity of Bob's prediction P) is non-local.

that of the Learned Prediction Experiment. Here, there is no good reason for Alice not to apply (C), in the sense that any theory claiming that he would reach the wrong conclusion must necessarily allow for signalling from Wigner to her. To see this, consider a slight variation of the experiment where at time t_W, which we assume to be very shortly before t_X, Wigner decides (based on the outcome of a random coin flip, for instance) whether or not to apply a destructive measurement to Bob's memory. If we could see a difference between Alice's prediction P' and her observed outcome X at time t_X, this would indicate signalling from Wigner to Alice, against special relativity. A more extreme case, resulting in signalling to the past, could be obtained by choosing t_W to be after t_X.

Example: Bohmian mechanics. Bohmian mechanics is a beautiful case study of Restriction 2 and its relation to non-signalling. Bohmian theory allows for a consistent analysis of the FR experiment [6], by violating assumption (Q), because Bohmian theory is only applicable to the experiment as a whole, but not to subsystems chosen subjectively by different observers [5]. See Supplementary Figure 1 for a quick depiction of the setup of the FR experiment, without calculation details. In the Bohmian analysis, Bob's prediction about outcome w, which he derives using rules (Q) and (C), is compromised by a destructive measurement, which is later applied to another agent, Alice [6]. In other words, the correctness of Bob's prediction for a value measured by Wigner depends on an operation applied to Alice. Hence, at the level of the physical explanation, an event in Alice's spacetime region affects Bob's spacetime region (including Wigner's measurement outcome w). These two regions may be spacelike separated. Such a non-local effect is not unexpected, since Bohmian theory is manifestly non-local. However, in this particular case, the non-locality is not restricted to hidden variables, but involves values known to agents, namely the outcome w of Wigner's measurement, as well as the prediction that Bob made for w. Note that, nonetheless, in the FR experiment, actual signalling from Alice's region to Bob's is not possible because the value w is the result of a destructive measurement of Bob. Hence,

Bob cannot verify the correctness of his own prediction for w. Neither can Wigner compare w to Bob's prediction, for he has no access to the latter.

Details on Objection 3: Restrictions on (C) impair reasoning. A pertinent question is whether Alice needed to use the reasoning assumption (C) to reach her conclusion that "$X = p$ holds with certainty." Did she need to think of p as indicative of Bob's prediction and then use (C) to combine her knowledge with Bob's? Alternatively, Alice could have modelled Bob and his lab as a quantum system (much like Wigner does). If she could simulate the quantum evolution of Bob's lab and memory, Alice would have concluded that at $t_{P'}$ the reduced state of the two quantum systems in her lab was classically correlated, $\rho_{PA} = \frac{1}{2}(|0\rangle\langle 0|_P \otimes |0\rangle\langle 0|_A + |1\rangle\langle 1|_P \otimes |1\rangle\langle 1|_A)$. This would allow Alice to predict that "$X = p$ holds with certainty" without using assumption (C).

This procedure, however, requires the agents to have complete information about each other's experiments to run reliable quantum simulations that include the others' reasoning processes. We assumed that the agents have such complete information for the Learned Prediction Experiment, thus enabling the alternative reasoning by Alice as described above. However, in real-life experiments, the agents generally have partial information only about the experiments carried out by others. In this case, we cannot let go of assumption (C): It is necessary for agents to combine each other's information, compressing their conclusions irrespective of the details of how they reached them (for example, through additional quantum experiments not known to everyone).

Furthermore, even if agents had access to a complete description of everything, they would also need the computational resources necessary to run simulations of other agents' knowledge. While simulations may be simplified, the classical resources required to reach predictions without (C) generally scale exponentially in the number of agents and steps of the experiment. This is already the case if knowledge is captured by classical probabilities, for keeping track of the joint probability distribution of N random variables requires memory of size exponential in N.

Assumption (C) thus plays a crucial role: it allows an agent to reach correct conclusions by treating others as abstract rational agents. More generally, we may say that a physical theory that allows for such an abstraction is 'classically compressible.' Classical compressibility is crucial for the scientific endeavour. The statements a physicist derives are usually based (directly or indirectly) on the results of experiments carried out before them by others, e.g., those who built and tested the devices used. However, if the physicist's theory was not classically compressible, they could not build upon these results.

Comment on the Bohmian mechanics analysis presented in [6]. The authors of [6] claim that Bohmian theory satisfies the three assumptions (Q), (C), and (S); as their analysis avoids the contradiction in the FR experiment, they conclude that Theorem 1 [3] must be false. This is due to a misunderstanding of the scope of assumption (Q), which was perhaps not clear in early versions of [3]: (Q) demands that an agent can apply the laws of standard quantum theory (notably, the Born rule) to *any* subsystem around the agent. In particular, according to this rule (Q), different agents may make different choices for the Heisenberg cut. Bohmian theory is incompatible with this assumption because its laws are only applicable to the universe as a whole rather than to subsystems. When applied to the FR thought experiment, the conclusions drawn from Bohmian analysis are at odds with those that follow from the standard postulates of quantum theory (e.g. [7]) applied to subsystem S as modelled by Alice. Hence, rule (Q) is violated by Bohmian theory.

Comment on the framework presented in [8]. The authors of [8] address the challenge of proposing reasoning rules that meet the desiderata described in the main text. To this aim, they introduce a framework that allows agents to keep track of their choices of Heisenberg cuts. Reasoning rule (C) is then *de facto* restricted by the requirement that an agent can incorporate knowledge of another agent only if the agents' choices of Heisenberg cuts are compatible. This allows them to meet the consistency and physicality desiderata in the scenarios explored. Whether the other desiderata can be met with this approach is not clear, because their framework assumes that all agents have access to a complete

description of the experimental setup as seen from the outside, which is not the case in situations where agents have partial information only.

References

[1] Nurgalieva, N., Mathis, S., del Rio, L. & Renner, R. Thought experiments in a quantum computer (2022). URL https://arxiv.org/abs/2209.06236.

[2] Polychronakos, A. P. Quantum mechanical rules for observed observers and the consistency of quantum theory (2022).

[3] Frauchiger, D. & Renner, R. Quantum theory cannot consistently describe the use of itself. *Nature Communications* **9**, 3711 (2018). URL https://doi.org/10.1038/s41467-018-05739-8.

[4] Nurgalieva, N. & del Rio, L. Inadequacy of modal logic in quantum settings. *Electronic Proceedings in Theoretical Computer Science* **287**, 267–297 (2019). URL https://doi.org/10.4204%2Feptcs.287.16.

[5] Nurgalieva, N. & Renner, R. Testing quantum theory with thought experiments. *Contemporary Physics* **61**, 193–216 (2020). URL https://doi.org/10.1080/00107514.2021.1880075.

[6] Lazarovici, D. & Hubert, M. How quantum mechanics can consistently describe the use of itself. *Scientific Reports* **9**, 470 (2019). URL https://doi.org/10.1038/s41598-018-37535-1.

[7] Nielsen, M. A. & Chuang, I. L. *Quantum Computation and Quantum Information* (Cambridge University Press, 2010).

[8] Vilasini, V. & Woods, M. P. A general framework for consistent logical reasoning in Wigner's friend scenarios: subjective perspectives of agents within a single quantum circuit (2022). URL https://arxiv.org/abs/2209.09281.
